# Identification of suicidality in patients with major depressive disorder via dynamic functional network connectivity signatures and machine learning

**DOI:** 10.1038/s41398-022-02147-x

**Published:** 2022-09-12

**Authors:** Manxi Xu, Xiaojing Zhang, Yanqing Li, Shengli Chen, Yingli Zhang, Zhifeng Zhou, Shiwei Lin, Tianfa Dong, Gangqiang Hou, Yingwei Qiu

**Affiliations:** 1 Department of Radiology, The Third Affiliated Hospital of Guangzhou Medical University, Guangzhou Medical University, Duobao AVE 56, Liwan district, Guangzhou, People’s Republic of China; 2 Guangdong Provincial Key Laboratory of Genome Stability and Disease Prevention and Regional Immunity and Diseases, Department of Pathology, Shenzhen University School of Medicine, Shenzhen, Guangdong, 518060 People’s Republic of China; 3 Department of Radiology, Huazhong University of Science and Technology Union Shenzhen Hospital, Shenzhen, 518000 People’s Republic of China; 4 Department of Psychiatry, Shenzhen Kangning Hospital, Shenzhen Mental Health Center, Shenzhen, 518020 People’s Republic of China; 5 Department of Radiology, Shenzhen Kangning Hospital, Shenzhen Mental Health Center, Shenzhen, 518020 People’s Republic of China

**Keywords:** Predictive markers, Depression, Pathogenesis

## Abstract

Major depressive disorder (MDD) is a severe brain disease associated with a significant risk of suicide. Identification of suicidality is sometimes life-saving for MDD patients. We aimed to explore the use of dynamic functional network connectivity (dFNC) for suicidality detection in MDD patients. A total of 173 MDD patients, including 48 without suicide risk (NS), 74 with suicide ideation (SI), and 51 having attempted suicide (SA), participated in the present study. Thirty-eight healthy controls were also recruited for comparison. A sliding window approach was used to derive the dFNC, and the K-means clustering method was used to cluster the windowed dFNC. A linear support vector machine was used for classification, and leave-one-out cross-validation was performed for validation. Other machine learning methods were also used for comparison. MDD patients had widespread hypoconnectivity in both the strongly connected states (states 2 and 5) and the weakly connected state (state 4), while the dysfunctional connectivity within the weakly connected state (state 4) was mainly driven by suicidal attempts. Furthermore, dFNC matrices, especially the weakly connected state, could be used to distinguish MDD from healthy controls (area under curve [AUC] = 82), and even to identify suicidality in MDD patients (AUC = 78 for NS vs. SI, AUC = 88 for NS vs. SA, and AUC = 74 for SA vs. SI), with vision-related and default-related inter-network connectivity serving as important features. Thus, the dFNC abnormalities observed in this study might further improve our understanding of the neural substrates of suicidality in MDD patients.

## Introduction

Major depressive disorder (MDD) is a very common mood disorder affecting more than 350 million people worldwide [1]. It is characterized by a persistent feeling of sadness or/and loss of pleasure [2]. MDD is a major risk factor for suicide, and 7% of men and 4% of women with MDD die from suicide [3]. Suicide has been reported to occur in a three-step gradual process consisting of suicidal ideations (SIs), suicidal attempts (SAs), and suicidal death (SD) [4–6]. Approximately 23% of those who have committed suicide had previously attempted it [7]. Therefore, the identification of patients with SI and SA may be an important intervention point for predicting and preventing suicide in MDD patients. However, the assessment of suicidality in patients with MDD is a difficult task due to the subjectivity of psychological scales and the unwillingness of patients to disclose their thinking or the acts they have committed [8–10]. Thus, it is urgent to find an objective biomarker to identify suicidality in MDD patients.

In recent decades, resting-state functional magnetic resonance imaging (rs-fMRI) has been widely used to investigate the suicidality of MDD patients based on the brain connectome [11–15] and regional brain activity [16–18], and it has presented inspiring results. For example, with the bilateral amygdala as a region of interest, Kang et al. reported that SA patients had significantly increased functional connectivity (FC) between the left amygdala and the right insula, as well as the left superior orbitofrontal area, and between the right amygdala and the left middle temporal area, compared with those who had not attempted suicide. Furthermore, they revealed a significant correlation between SI scores and the FC of the right amygdala with the right parahippocampal area in the SA group [12]. Using the whole-brain FC analysis method, Chen et al. found that SA patients had significantly higher FC strength in the right orbitofrontal cortex and the bilateral dorsomedial prefrontal cortex than non-SA patients, which is thought to be associated with a higher risk of suicidal behavior in MDD patients [15]. Meanwhile, using the amplitude of low-frequency fluctuation (ALFF) method, Fan et al. found that depressed patients with SA had increased activity at a low frequency (0.01–0.08 Hz) in the right primary auditory cortex (superior temporal gyrus) compared with the depression group without SA and the healthy control group [17]. Although these studies have provided important preliminary results, they also suffer from critical limitations. For example, these studies evaluated brain activity or connectivity by averaging the time series within the entire scanning period, which ignored the time-varying neural activity during the scanning period; thus, the results are far from conclusive.

More recently, dynamic functional network connectivity (dFNC) has been developed to analyze dynamic functional coordination between different parts of the nervous system in the human brain and to uncover the hidden dynamic information embedded in the resting state [19–23]. One can consider dFNC to be more specific than static FC because it unpacks temporal features otherwise averaged in static FC measures [24–26]. dFNC has been successfully used to investigate the dynamic functional modulations in patients with schizophrenia [20], patients with hepatic encephalopathy [21], and healthy aged subjects [22]. However, no study has been conducted to uncover the relationship between dFNC and suicidality in MDD.

The purpose of this study was (1) to identify suicidality-related modulations of dynamic functional coordination in MDD patients with different suicide risk levels using resting-state functional magnetic resonance imaging (rsMRI) and the dFNC method, and (2) to investigate whether this dynamic information can be used to screen MDD patients from healthy controls or even to distinguish MDD patients with varying suicide risk levels.

## Materials and methods

### Subjects

MDD patients were recruited from the Department of Depression at Shenzhen Kangning Hospital. They received a diagnosis of MDD according to the DSM-IV criteria [27–30]. Other inclusion criteria were as follows: (1) score ≥ 17 on the 17-item version of the Hamilton Rating Scale for Depression (HAMD) [31]; (2) Chinese Han nationality; (3) age between 18 and 60 years; and (4) right-handedness. Patients were excluded from the study if they had any other psychological disorder except MDD or had a history of drug or alcohol abuse or dependence; prior significant neurological or medical illness, including substantial head trauma; severe physical illness or infectious disease; or contraindications for MRI. Thereafter, MDD patients were further sorted into three subgroups according to a system used in previous research [32, 33]. These groups included 51 SA patients, defined as those having committed a documented self-injurious act with the intent to die [32, 34]; 74 SI patients, defined as those thinking about engaging in an act designed to end their life but who have not attempted it [33]; and 48 NS patients, defined as having no SAs or SIs. In addition, 38 age-, gender-, and education-matched healthy controls (HCs) were recruited from the community with the same criteria as MDD patients, except that HCs did not have any personal or family history of significant psychiatric disorders or other systemic diseases.

The study protocol was approved by the Research Ethics Committee of Shenzhen Kangning Hospital (No. 2018-S017). All the patients provided written informed consents in compliance with the code of ethics of the World Medical Association (Declaration of Helsinki).

### MRI data acquisition and pre-processing

rs-fMRI was obtained using a 3.0-Tesla scanner (Discovery MR750 System; General Electric) with an eight-channel head coil. During the scanning, each participant was asked to keep still with their eyes closed, but not to fall asleep and not to think about anything. rs-fMRI was collected using an echo-planar imaging (EPI) sequence with the following parameters: TR/TE = 2000/30 ms; flip angle = 90°; thickness/gap = 3.5/0 mm; acquisition matrix = 64 × 64; field of view (FOV) = 224 mm^2^; 33 axial slices; and 240 time points (8 min).

The preprocessing of rs-fMRI data was performed using the Statistical Parametric Mapping 12 (SPM12) software package and the Data Processing Assistant for Resting-State fMRI (DPARSF) [35] as in our previous work [21, 36]. The main steps were as follows: (1) we removed the first 10 time points to allow for signal equilibration; (2) we performed slice-timing correction and motion correction; and (3) we applied spatial normalization to the Montreal National Institute space (MNI) and smoothing using a 6 mm full-width at high maximum (FWHM) Gaussian kernel. Individuals were excluded from further analysis if their maximum head motion exceeded 2.5 mm in displacement or 2.5° in rotation.

### Group independent component analysis

We analyzed the processed fMRI data using a group-level spatial independent component analysis (ICA) in the GIFT package (version 3.0b) (http://mialab.mrn.org/software/gift/index.html). First, a two-stage principal component analysis was conducted to decrease computational complexity. The functional data were accurately dimension-reduced temporally, and then the reduced data from all of the subjects were concatenated into a single dataset along the temporal dimension and passed through another dimension reduction. Second, we applied the Infomax algorithm [37] to decompose the grouped data into 32 automatically estimated independent components (ICs). In this step, we generated the spatial map and the time course of the BOLD signal for each IC. To ensure the stability of decomposition, we repeated the GICA infomax algorithm 100 times using ICASSO (http://research.ics.aalto.fi.ica/icasso) [38]. Finally, the ICs for each participant were derived from a group ICA back reconstruction step and were Fisher-transformed to *z* values.

Next, we eliminated nine ICs because of noise impact and the low-frequency/high-frequency ratio [39]. The remaining 23 ICs were sorted to eight large-scale networks based on their anatomical and functional properties: the auditory network (AUD), visual network (VIS), sensorimotor network (SMN), dorsal attention network (DAN), ventral attention network (VAN), limbic network (LN), frontoparietal network (FPN), and default mode network (DMN) [40, 41].

### dFNC computation

We estimated the dFNC by computing Pearson’s correlations between time courses of ICs using the sliding window method. We set a window size of 50 TRs (100 s) with a step size of 1 TR (3 s) for each participant in accordance with previous studies [42–44], which resulted in a total of 180 23 × 23 FNC matrices for each subject. The graphical least absolute shrinkage and selection operator (LASSO) algorithm was used to regularize the matrices [45]. Then, we applied the K-means algorithm with the sqEuclidean function to divide the dFNC windows into a set of separate clusters [46, 47]. We repeated the clustering algorithm 500 times to increase the chances of escaping the local minima [48]. Finally, we computed the dFNC properties, including mean dwell time (DT), fraction time (FT), and number of transitions (NT).

### Statistical analysis

We performed the Kolmogorov–Smirnov test to indicate normally distributed data for the majority demographic and clinical characteristics. Two-sample *t* tests and one-way analysis of variance (ANOVA) were performed to compare continuous variables, and the Chi-square test was performed to detect intergroup differences in categorical variables. In addition, we performed two-sample *t* tests to examine the group effect on the dFNC parameters for each state, including the functional connectivity, the mean DT, the FT, and the NT. Statistical significance was considered at *P* < 0.05 (two-tailed), with FDR correction.

### Feature selection and classification model construction

We used the *F*-score for feature selection, which is a simple and generally quite effective method, as documented previously [49]. We used the pairwise classification method of support vector machine (SVM) to build up models for distinguishing MDD from HC and for further differentiating MDD patients with different suicide risk levels (NS vs. SI, NS vs. SA, and SA vs. SI) in the LIBSVM toolbox (http://www.csie.ntu.edu.tw/cjlin/libsvm/) based on MATLAB (MathWorks, Natick, MA). Since not all of the subjects had experienced all states, only the subset of subjects within a given state was used for classification. First, the *F-*score of each feature (connection) in the training set was calculated and ranked in descending order as in previous studies [50]. Second, a subset of the original training set was generated by including the features (connections) with the top *N* *F-*scores successively, where *N* = 1, 2,…, *m*, and *m* is the total number of features (connections) (23 × 22/2) [51]. Then, a grid search using leave-one-out cross-validation (LOOCV) was carried out to find the optimized values of (C, γ), where C denotes the penalty parameter, and γ represents the kernel width parameter [52]. Thereafter, the optimized values of (C, γ) were used to construct the SVM classifier, which was subsequently used to predict labels in the test subset. The area under the curve (AUC), accuracy, sensitivity, and specificity were obtained to evaluate the performance of the classifier [53]. A permutation test was performed to determine whether the obtained accuracy rate was significant (*P* < 0.05). For comparison, the set of selected dFNC features was also fed to other machine learning algorithms, which included random forest, Bayesian, and deep learning algorithms. These classification algorithms are available in Python using the scikit library [54].

## Results

### Demographics and clinical characteristics

The details of demographic and clinical characteristics are shown in Table 1. We did not observe any significant differences in age, education level, gender, or head motion between MDD patients and HCs (*P* > 0.05) or among the MDD subgroups.

**Table 1:** Demographic and clinical characteristics of HCs and MDD with different suicide risks.

Demographic characteristics	MDD (*n* = 173)	SA (*n* = 51)	SI (*n* = 74)	NS (*n* = 48)	HCs (*n* = 38)	*P* Value (MDD vs.HC)	*P* Value (Subgroups of MDD)	*P* Value (subgroups of MDD vs. HC)
Gender(male/female)	57/116	12/39	25/49	20/28	13/25	0.88	0.30	0.22
Age(y)	35 ± 13	33 ± 15	35 ± 12	39 ± 13	37 ± 13	0.46	0.10	0.15
Education(y)	14 ± 3	14 ± 3	14 ± 3	13 ± 4	15 ± 4	0.07	0.19	0.30
Depression severity (HAMD)	23 ± 5	23 ± 5	24 ± 5	22 ± 5	–	–	0.13	–
Anxiety severity (HAMA)	17 ± 7	16 ± 7	19 ± 6	17 ± 7	–	–	0.20	–
Head motion	0.09 ± 0.07	0.08 ± 0.06	0.10 ± 0.06	0.09 ± 0.07	0.10 ± 0.08	0.15	0.59	0.31

*MDD* major depressive disorder, *SA* suicide attempter, *SI* suicide ideation, *NS* neither *SA* nor *SI,* *HCs* healthy controls, *HAMD* Hamilton Depression Scale, *HAMA* Hamilton Anxiety Scale.

### dFNC states and properties

**Fig. 1:**
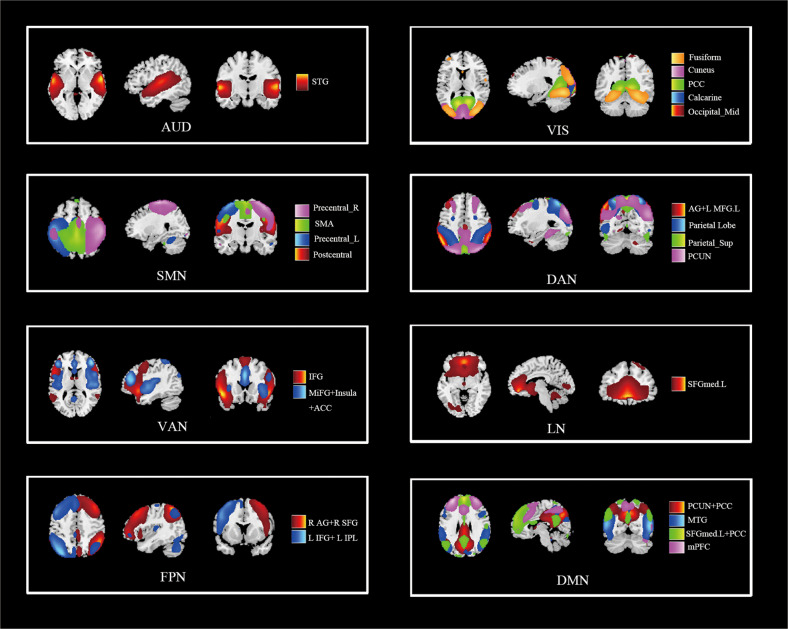
Spatial maps of 23 independent components sorted into eight intrinsic networks overlaid on the Montreal Neurological Institute (MNI) template. Color bar represents the independent component (IC). AUD auditory network, VIS visual network, SMN somatomotor network, DAN dorsal attention network, VAN ventral attention network, LN limbic network, FPN frontoparietal network, DMN default mode network, L left, R right, STG superior temporal gyrus, Fusiform, fusiform gyrus, Cuneus cuneus cortex, PCC posterior cingulate cortex, Calcarine calcarine cortex, Occipital_Mid middle occipital gyrus, Postcentral postcentral gyrus, Precentral precentral gyrus, SMA supplementary motor area, AG angular gyrus, MFG middle frontal gyrus, Parietal_Sup superior parietal gyrus, PCUN precuneus, IFG inferior frontal gyrus, MiFG middle inferior frontal gyrus, lnsula insular cortex ACC anterior cingulate cortex, SFGmed superior frontal gyrus, medial, SFG superior frontal gyrus, IPL inferior parietal lobule, MTG middle temporal gyrus, mPFC medial prefrontal cortex.

**Fig. 2:**
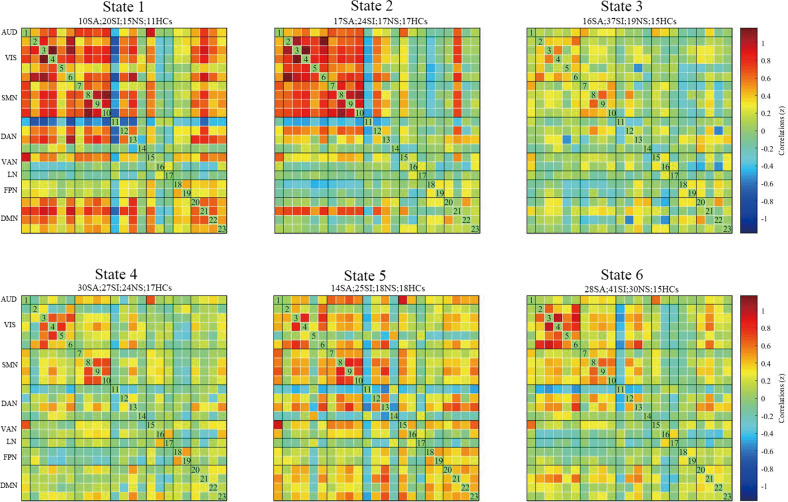
State plots and the number of members in each group with each state. Whole-brain cross-correlation matrices of states 1–6 are shown. The number of participants who entered each state is indicated above the state plots. SA suicide attempter, SI suicide ideation, NS neither SA nor SI, HCs healthy controls, AUD auditory network, VIS visual network, SMN somatomotor network, DAN dorsal attention network, VAN ventral attention network, LN limbic network, FPN frontoparietal network, DMN default mode network.

A total of 23 ICs were identified and categorized into eight large-scale networks (Fig. 1). Table 2 lists the ICs’ labels and peak activation coordinates. Finally, six functional states were determined by the cluster validity index and elbow criterion [55, 56] (Fig. 2). States 1, 2, and 5 represented the strongly connected states, which exhibited strong positive connectivity across SM, AUD, VIS, and DMN. State 1 was engaged by 11 HC, 15 NS, 20 SI, and 10 SA participants; state 2 was engaged by 17 HC, 17 NS, 24 SI, and 17 SA participants; state 5 was engaged by 18 HC, 18 NS, 25 SI, and 14 SA participants. States 3, 4, and 6 exhibited weaker connectivity among all networks compared with states 1, 2, and 5. State 3 was engaged by 15 HC, 19 NS, 37 SI, and 16 SA participants; state 4 was engaged by 17 HC, 24 NS, 18 SI, and 30 SA participants; state 6 was engaged by 15 HC, 30 NS, 41 SI, and 28 SA participants. Among all of these states, state 3 had the highest frequency and the longest average residence time.

**Table 2:** Independent components sorted into functional domains.

Functional domains and resting-state network		IC number	MIN peak coordinate		
			*X*	*Y*	*Z*
AUD	STG	001	62.5	−5.5	0.5
	Fusiform	011	−23.5	−99.5	−3.5
	Cuneus	013	0.5	−90.5	2.5
VIS	PCC	016	15.5	−57.5	6.5
	Calcarine	028	2.5	−84.5	36.5
	Occipital_Mid	029	26.5	−72.5	−14.5
SMN	Postcentral	003	59.5	−3.5	24.5
	Precentral_L	005	−32.5	−24.5	71.5
	SMA	006	0.5	−44.5	71.5
	Precentral_R	008	35.5	−21.5	71.5
DAN	AG + MFG.L	026	−54.5	−59.5	41.5
	Parietal Lobe	027	23.5	−69.5	60.5
	Parietal_Sup	031	0.5	−65.5	62.5
	PCUN	032	41.5	−77.5	35.5
VAN	IFG	015	−50.5	15.5	−6.5
	MiFG + lnsula + ACC	025	45.5	42.5	9.5
LN	SFGmed.L	020	−3.5	45.5	−14.5
FPN	R AG + R SFG	009	44.5	−60.5	53.5
	L IFG + L IPL	019	−35.5	−69.5	53.5
DMN	PCUN + PCC	012	0.5	−74.5	38.5
	MTG	018	53.5	−68.5	5.5
	SFGmed.L + PCC	023	−0.5	59.5	18.5
	mPFC	024	−32.5	54.5	18.5

*IC* independent component, *AUD* auditory network, *VIS* visual network, *SMN* somatomotor network, *DAN* dorsal attention network, *VAN* ventral attention network, *LN* Limbic network, *FPN* frontoparietal network, *DMN* default mode network, *MNI* Montreal Neurological Institute, *L* left, *R* right, *STG* superior temporal gyrus, *Fusiform* fusiform gyrus, *Cuneus* cuneus cortex, *PCC* posterior cingulate cortex, *Calcarine* calcarine cortex, *Occipital_Mid* middle occipital gyrus, *Postcentral* postcentral gyrus, *Precentral* precentral gyrus, *SMA* supplementary motor area, *AG* angular gyrus, *MFG* middle frontal gyrus, *Parietal_Sup* superior parietal gyrus, *PCUN* precuneus, *IFG* inferior frontal gyrus, *MiFG* middle inferior frontal gyrus, *lnsula* insular cortex, *ACC* anterior cingulate cortex, *SFG*med superior frontal gyrus medial, *SFG* superior frontal gyrus, *IPL* inferior parietal lobule, *MTG* middle temporal gyrus, *mPFC* medial prefrontal cortex.

**Fig. 3:**
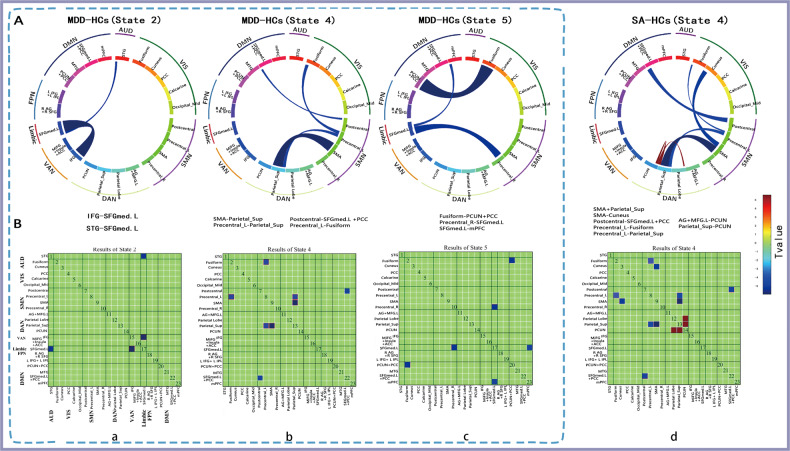
Functional network connectivity differences between patients with major depressive disorder (MDD) and HCs in state 2, state 4, and state 5 and between SAs and HCs in state 4. Significance was corrected using the false-discovery rate (FDR) over the total of 253 (23 × 22/2) dFNC values in each state. The circles indicate significant (*P* < 0.05, FDR-corrected) *t* tests. A wider line means a large group difference. Red lines represent increased connectivity, while blue lines represent decreased connectivity between two groups. AUD auditory network, VIS visual network, SMN, somatomotor network, DAN dorsal attention network, VAN ventral attention network, LN limbic network, FPN frontoparietal network, DMN default mode network, L left, R right, STG superior temporal gyrus, Fusiform fusiform gyrus, Cuneus, cuneus cortex, PCC posterior cingulate cortex, Calcarine calcarine cortex, Occipital_Mid middle occipital gyrus, Postcentral postcentral gyrus; Precentral precentral gyrus, SMA supplementary motor area, AG angular gyrus, MFG middle frontal gyrus, Parietal_Sup superior parietal gyrus, PCUN precuneus, IFG inferior frontal gyrus, MiFG middle inferior frontal gyrus, lnsula insular cortex, ACC anterior cingulate cortex, SFGmed superior frontal gyrus medial, SFG, superior frontal gyrus, IPL inferior parietal lobule, MTG middle temporal gyrus, mPFC medial prefrontal cortex.

Intergroup comparison revealed that MDD patients exhibited lower connections involved in both the strongly connected (state 2 and state 5) and the weakly connected (state 4) states compared with HCs (Fig. 3a–c). Specifically, MDD patients had inter-network disconnectivity between the LN and the VAN and between the LN and the AUD in state 2 (Fig. 3a); had reduced inter-network FC of the SMN and the VIS, of the SMN and the DMN, and of the SMN and the DAN in state 4 (Fig. 3b); and had attenuated inter-network FC of the DMN and the VIS, of the DMN and the LN, and of the SMN and the LN in state 5 (Fig. 3c). Moreover, we found that MDD related weaker connectivity within the weakly-connectivity state was mainly driven by SA, given that only SA demonstrated significantly altered connections in state 4 compared with HCs, with connections involving the SMN–VIS, SMN–DMN, and SMN–DAN, and within the DAN (Fig. 3d) being altered. No differences were found between other subgroups of MDD patients and HCs.

### Other dFNC indices

MDD patients had more DT than HCs in state 6 (weakly connected state). Further comparison between MDD subgroups and HCs revealed that the NS group showed more DT in state 6 (weakly connected state), while the SA group had less DT in state 5 (strongly connected state) compared with HCs (Supplementary Fig. 1). No significant difference in FT and NT was found between MDD patients and HCs (Supplementary Fig. 2).

Among the MDD subgroups, the SA group had more DT in state 4 (weakly connected state) than the NS and SI groups and less DT in state 5 (strongly connected state) than the SI group (Supplementary Fig. 3). There was no significant difference in FT and NT among the subgroups (Supplementary Fig. 2).

### Classification of SVM

**Fig. 4:**
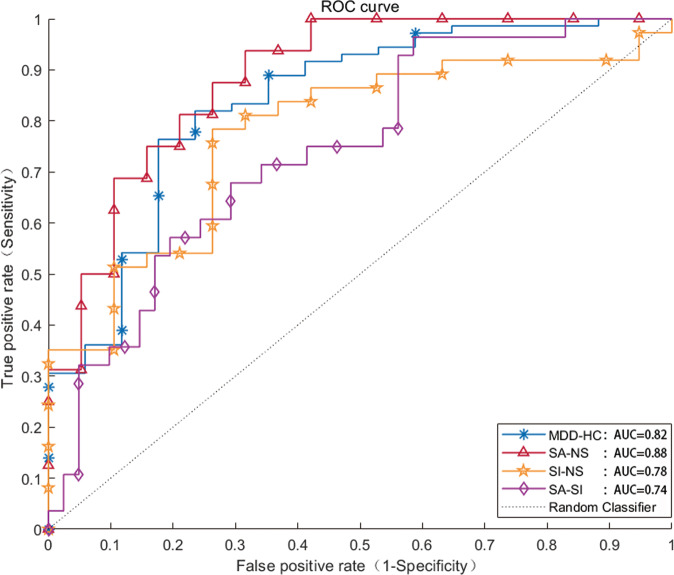
ROC of the best classifiers between groups. AUC area under the curve, MDD major depressive disorder, SA suicide attempter, SI suicide ideation, NS individuals had neither SA nor SI, HCs healthy controls.

**Fig. 5:**
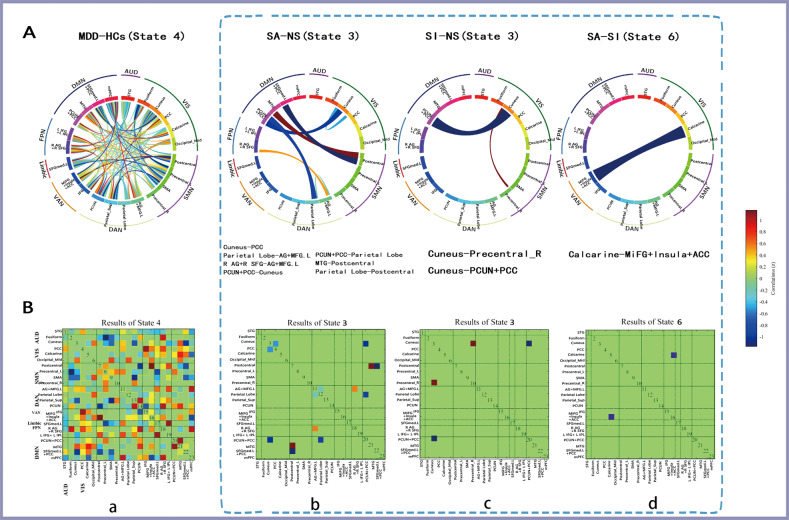
Consensus functional connections in distinguishing patients with major depressive disorder (MDD) from HCs and in distinguishing suicidality among MDD patients. The brain region of each cluster is represented by a square on the circumference of the big circle. The lines connecting two squares represent the connections between the corresponding two brain regions. The thickness of the line represents the support vector classification weight of the connection. The thicker the line, the larger the weight. Red lines represent positive weight, while blue lines represent negative weight. AUD auditory network, VIS visual network, SMN somatomotor network, DAN dorsal attention network, VAN ventral attention network, LN limbic network, FPN frontoparietal network, DMN default mode network, L left, R right, STG superior temporal gyrus, Fusiform fusiform gyrus, Cuneus cuneus cortex, PCC posterior cingulate cortex, Calcarine calcarine cortex Occipital_Mid middle occipital gyrus, Postcentral postcentral gyrus, Precentral precentral gyrus, SMA supplementary motor area, AG angular gyrus, MFG middle frontal gyrus, Parietal_Sup superior parietal gyrus, PCUN precuneus, IFG inferior frontal gyrus, MiFG middle inferior frontal gyrus, lnsula insular cortex, ACC anterior cingulate cortex, SFGmed superior frontal gyrus medial, SFG superior frontal gyrus, IPL inferior parietal lobule, MTG middle temporal gyrus, mPFC medial prefrontal cortex.

In the classification of MDD and HCs, the model constructed with state 4 (weakly connected state) had the most powerful discrimination efficiency (AUC = 0.82; ACC = 86.73; sensitivity = 0.78; specificity = 0.76) (Table 3 and Fig. 4). In this model, a total of 197 connections contributed to the classification (Fig. 5). In stratifying MDD patients with different suicidal risk levels, models constructed with state 3 (a weakly connected state) had the best discrimination efficiency in classifying SA from NS (AUC = 0.88; ACC = 80; sensitivity = 0.88; specificity = 0.74) (Table 3 and Fig. 4) and in classifying SI from NS (AUC = 0.78; ACC = 75; sensitivity = 0.78; specificity = 0.74) (Table 3 and Fig. 4), with the VIS-, DMN-, and DAN-related intra-network and inter-network serving as important features (Fig. 5). We also found that state 6 (weakly connected state) performed best in classifying the SA and SI groups, with an AUC of 0.74, an ACC of 68.12, a sensitivity of 0.68, and a specificity of 0.71 (Table 3 and Fig. 4), with the inter-network FC of VAN–VIS serving as an important feature (Fig. 5). Models constructed with SVM also performed well in distinguishing MDD subgroups from HCs; details can be found in Supplementary Fig. 4, Supplementary Fig. 5, and Supplementary Table 1.

**Table 3:** Classification performance for linear SVM in each state between MDD and HCs and among MDD subgroups.

Groups	State	Acc	AUC	Sensitivity, %	Specificity, %	Cut off Point	Number	*acc*-*P*
MDD-HCs	State 1	80.36	0.12	0.18	0.64	0.14	1	0.994
	State 2	81.33	0.72	0.64	0.76	0.49	9	0.023*
	State 3	82.76	0.31	0.42	0.47	0.19	1	1
	State 4	86.73	0.82	0.78	0.76	0.59	156	0.001*
	State 5	80	0.74	0.61	0.83	0.51	15	0.02*
	State 6	86.84	0.57	0.60	0.60	0.36	1	1
SA-NS	State 1	64	0.71	0.50	0.93	0.47	23	0.17
	State 2	64.71	0.58	0.71	0.65	0.46	1	0.172
	State 3	80	0.88	0.88	0.74	0.64	13	0.012*
	State 4	79.63	0.81	0.83	0.75	0.63	99	0.008*
	State 5	75	0.80	0.86	0.67	0.57	6	0.035*
	State 6	72.41	0.75	0.71	0.77	0.55	1	0.018
SI-NS	State 1	62.86	0.58	0.60	0.73	0.44	6	0.189
	State 2	63.41	0.66	0.71	0.59	0.42	3	0.19
	State 3	75	0.78	0.78	0.74	0.58	2	0.023*
	State 4	62.75	0.61	0.70	0.58	0.41	99	0.15
	State 5	72.09	0.72	0.68	0.78	0.53	46	0.027*
	State 6	50.70	0.07	0.17	0.2	0.04	1	0.56
SA-SI	State 1	66.67	0.48	0.4	0.85	0.34	202	0.16
	State 2	63.41	0.61	0.88	0.42	0.37	4	0.194
	State 3	77.36	0.63	0.63	0.78	0.49	39	0.014*
	State 4	56.14	0.57	0.57	0.63	0.36	3	0.386
	State 5	69.23	0.57	0.43	0.8	0.34	10	0.124
	State 6	68.12	0.74	0.68	0.71	0.48	1	0.046*

Number, predictive accuracy as a function of the number of connections used in the best classification process.

*Acc* accuracy, *acc-P* the results of permutation test, *MDD* major depressive disorder, *SA* suicide attempter, *SI* suicide ideation, *NS* neither *SA* nor *SI*, *HCs* healthy controls.

*P < 0.05.

### Models constructed with other machine learning methods

Models constructed with deep learning and Bayesian algorithm exhibited comparable classification efficiencies when compared with the model constructed with the SVM, and also indicated that the weakly connected state (state 4) performed best in distinguishing MDD patients from HCs (AUC = 0.81 for deep learning; AUC = 0.74 for Bayesian algorithm). In contrast, models constructed with state 3 (weakly connected state) had the best discrimination efficiencies in distinguishing SA from SI (AUC = 0.87 for deep learning; AUC = 0.82 for Bayesian algorithm); models constructed with state 3 (deep learning) and state 5 (Bayesian algorithm), which are both weakly connected states, performed best in distinguishing SA from NS (AUC = 0.97 for deep learning; AUC = 0.87 for Bayesian algorithm); and models constructed with state 5 (a weakly connected state) performed best in distinguishing SI from NS (AUC = 0.88 for deep learning; AUC = 0.76 for Bayesian algorithm). The model constructed with random forest presented relatively weak efficiency. Details can be found in the Supplementary Materials (Supplementary Table 2 and Supplementary Fig. 6).

## Discussion

To the best of our knowledge, this is the first study to investigate suicidality in MDD patients using dFNC and machine learning algorithms. Our results demonstrated that MDD patients exhibited suicide risk-specific disruption in dFNC. Specifically, NS patients presented prolonged DT in a weakly connected state (state 6), while SA patients presented shortened DT in a relatively strongly connected state (state 5); connectivity matrix analysis revealed that MDD patients had widespread hypoconnectivity in both the strongly connected states and the weakly connected states, while the dysfunctional connectivity within the weakly connected state was mainly driven by the SA group. Furthermore, dFNC matrices, especially for the weakly connected state, can be used to distinguish MDD patients from healthy controls and even to identify suicidality of MDD patients, with the VIS-related and DMN-related inter-network connectivity serving as important features. Thus, the dynamic FNC abnormalities observed in this study might further improve our understanding of the neural substrates of suicidality in MDD patients.

As expected, we found that MDD patients had widespread FC attenuation in both the strongly and the weakly connected states, involving the intra-network and inter-network connectivity of the primary network (VIS, AUD, SMN) and the high-level cognitive network (DMN). The widespread dysconnectivity in MDD patients has been well-documented previously [56, 57]. Using static functional connectivity analysis, researchers have consistently reported that MDD has widespread attenuation of brain connectivity, mainly involving the DMN [58, 59], the VIS [60, 61], the SMN [62, 63], and the FPN [41]. With dFNC analysis, a recent study has also shown that MDD patients have widespread FC attenuation across both strongly and weakly connected states. Moreover, researchers have demonstrated that disrupted node properties within both strongly and weakly connected states correlate with the depressive symptom severity and cognitive performance of MDD patients [46, 56, 64]. The present findings are consistent with these studies. Furthermore, we found that MDD-related attenuated FC within the weakly connected state was mainly driven by SA patients, given that we did not find any significant differences in NS and SI patients compared with HCs. The exact mechanism for this finding is still unclear. The weakly connected state has been well-documented to be related to self-focused thinking [56, 65]; therefore, more DT and weaker FC within the weakly connected state in SA patients may represent more severe self-focused thinking than in HC and other MDD patients. Given that increased self-focused thinking is closely linked to suicidal behavior [66–69], it is reasonable to assume that weaker FC within the weakly connected state in SA patients may increase their vulnerability to suicidal behavior [46, 70, 71]. Taken together, our present findings supplement current knowledge by showing that MDD-related attenuated FC within the weak connectivity state is mainly driven by SA patients, which may underlie their suicidal behavior.

Interestingly, the classification model constructed with SVM also demonstrated that the weakly connected state performed better in distinguishing MDD patients from HCs and also in stratifying suicidal risk among MDD patients than the strongly connected state. This is not surprising, given that the weakly connected state has been closely linked to depression [56, 57, 72], and the intergroup differences between SA and NS in the present study also pointed to the weakly connected state. Our model, constructed with the weakly connected state for distinguishing MDD patients from HCs, is comparable with previous classification models constructed with cerebral functional features [73, 74] and is superior to models constructed with structural features [75, 76]. Moreover, our models also had powerful efficiency in stratifying patients with different suicidal risk levels, which supplements previous findings on using structural features to stratify MDD patients with diverse suicide risk [33, 77]. Applying a machine learning approach, Hong et al. found that structural MRI could correctly identify SA patients and SI patients, with an accuracy of 78.59% [77]. Our models constructed with SVM and the weakly connected state had similar classification power, indicating that dFNC may be an additional potential feature for stratifying MDD patients with diverse suicide risk levels.

Notably, the features that contributed to stratifying MDD patients with diverse suicide risk levels mainly involved the VIS-related and DMN-related inter-network connectivity within the weakly connected state. The VIS network plays an important role in facial expression recognition and visual information processing [78]. Dysfunction in the visual regions is significantly associated with MDD [79, 80], while a disproportional reduction in the amount of negative information held in visual working memory is correlated with high level of SI [81]. The DMN plays an important role in psychological processes related to suicidal behavior, such as controlling the vividness of negative mental imagery and improving self-referential processing [82]. Therefore, the discoordination of the VIS and the DMN with other large-scale networks may lead to increased negative information held in visual working memory and inability to control the vividness of negative mental imagery, which is subsequently involved in suicidal behavior in MDD patients. Our study provides a new perspective on the neurophysiological abnormalities of suicidality in MDD.

### Limitations

Limitations of our work include that the subjects in the present study were recruited from a single site; thus, the classification models constructed with the SVM and dFNC lack external validation, although we used LOOCV to compensate for this as in previous studies [83, 84]. Future studies should collect data from multiple sites and centers to validate these preliminary results. Another drawback is that we used single-mode imaging; features derived from a multimodal imaging approach (i.e., anatomical MRI, diffusion MRI, arterial spin labeling MRI) would perform better in stratifying MDD patients with different suicide risk levels than single-mode imaging [84, 85]. Therefore, multimodal imaging should be considered in the future to investigate diagnostic efficiency. Although structured interviews are commonly used and reliable evaluation methods to assess patients’ suicidal risk [86, 87], other suicidal risk assessment scales, such as the Nurses’ Global Assessment of Suicide Risk (NGASR), should be jointly used to assess the suicidality of MDD patients in the future [88, 89].

## Conclusion

In summary, MDD patients exhibit suicide risk–specific disruption in dFNC, which advances our understanding of the neuromechanisms of suicidality in MDD patients. We also established models to distinguish MDD patients from HCs and even to screen MDD patients with different suicidal risk levels. Thus, altered dFNC may emerge as a promising and quantifiable candidate marker of suicidal risk levels in patients with depression.

## Supplementary information

Supplementary Figure 1

Supplementary Figure 2

Supplementary Figure 3

Supplementary Figure 4

Supplementary Figure 5

Supplementary Figure 6

Supplementary Table 1

Supplementary Table 2
