# Supplementary figures and images for: Identification of suicidality in patients with major depressive disorder via dynamic functional network connectivity signatures and machine learning
Source: Transl Psychiatry. 2022 Sep 12;12:383. doi: 10.1038/s41398-022-02147-x (PMC9467986; doi:10.1038/s41398-022-02147-x)

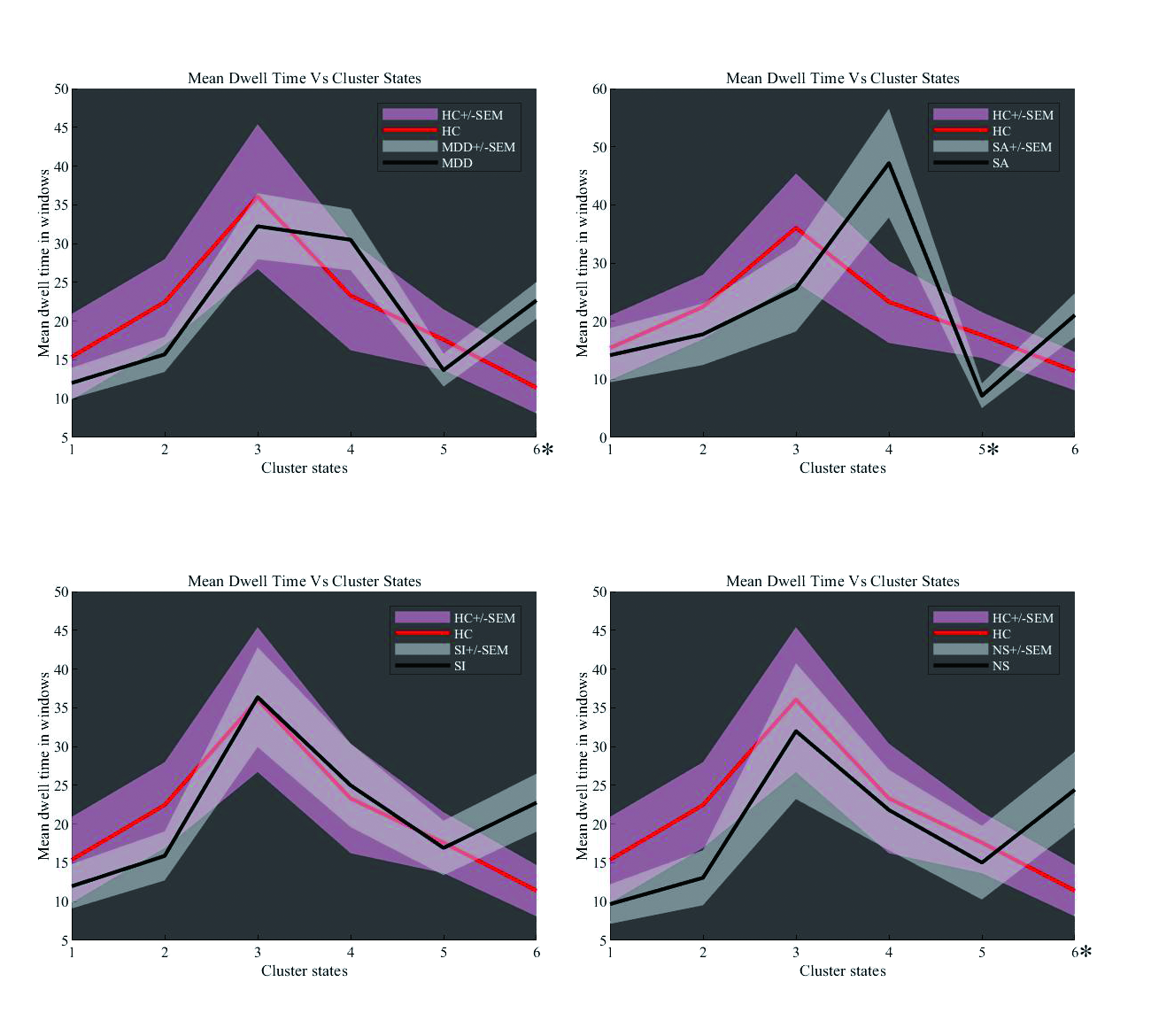

Supplement: Supplementary file 1 — Supplementary Figure 1 [file 41398_2022_2147_MOESM1_ESM.tif]

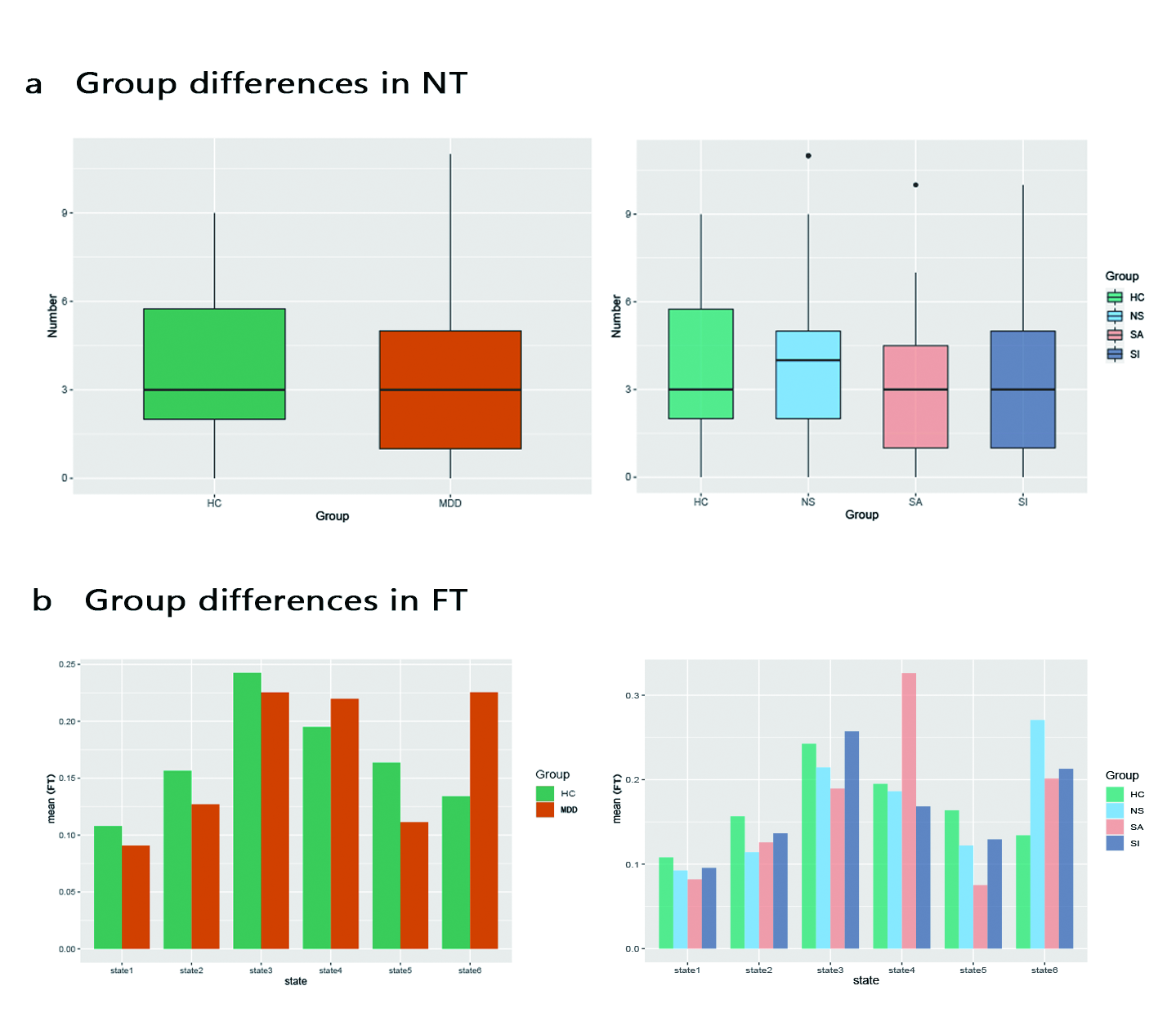

Supplement: Supplementary file 2 — Supplementary Figure 2 [file 41398_2022_2147_MOESM2_ESM.tif]

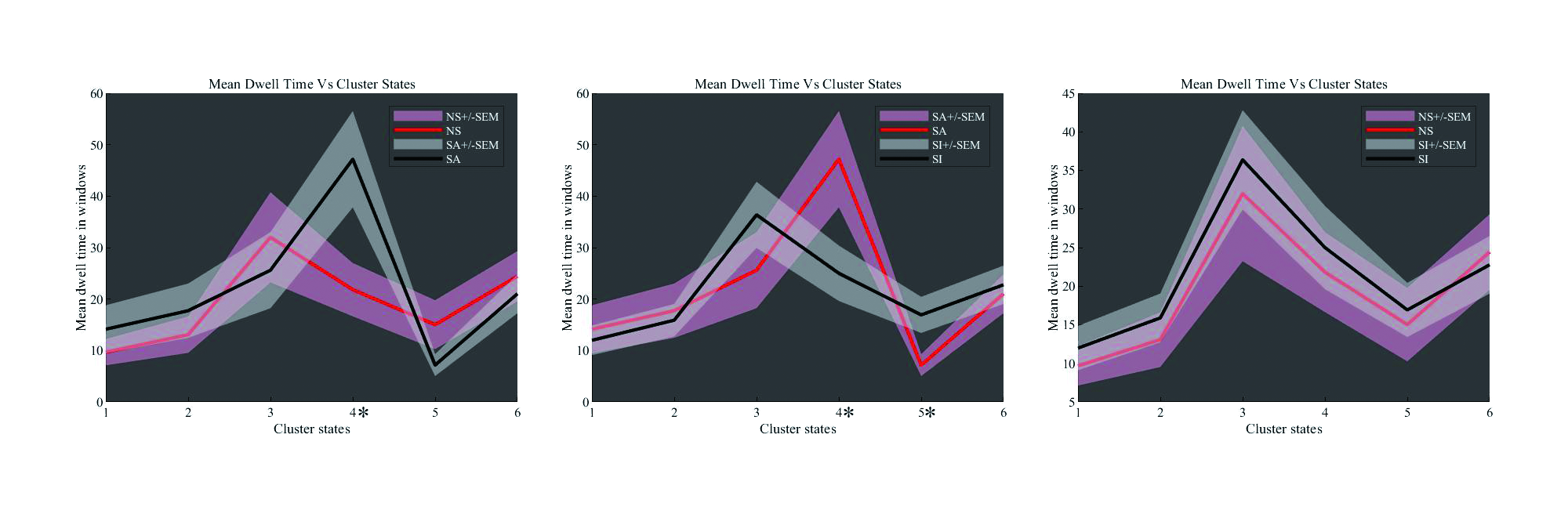

Supplement: Supplementary file 3 — Supplementary Figure 3 [file 41398_2022_2147_MOESM3_ESM.tif]

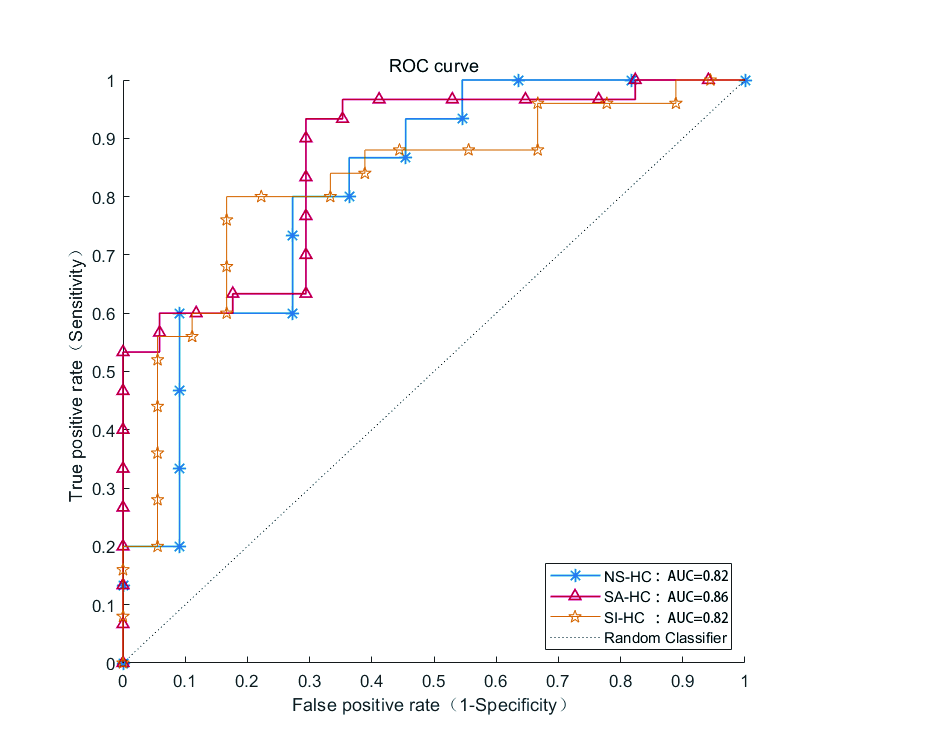

Supplement: Supplementary file 4 — Supplementary Figure 4 [file 41398_2022_2147_MOESM4_ESM.tif]

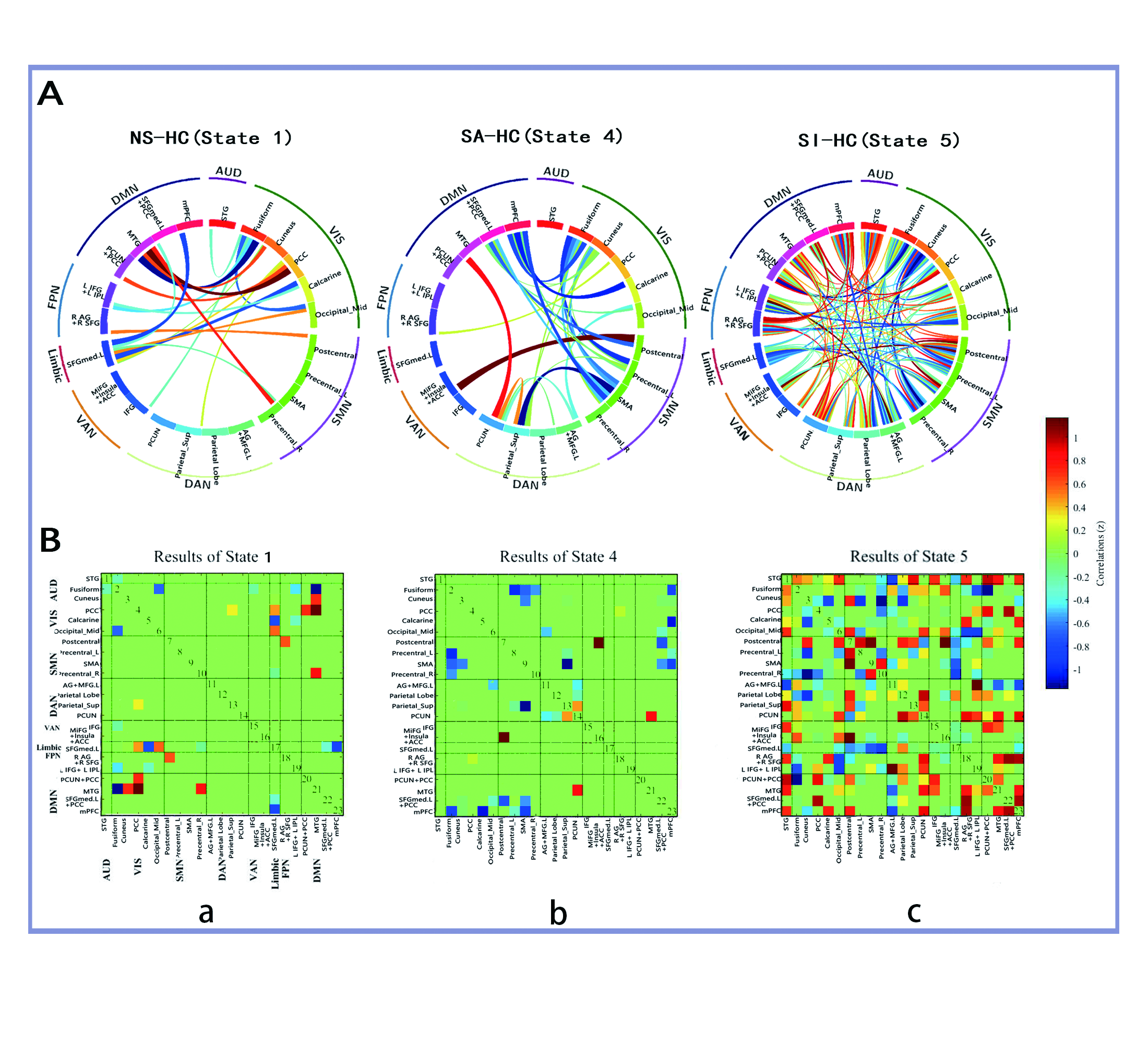

Supplement: Supplementary file 5 — Supplementary Figure 5 [file 41398_2022_2147_MOESM5_ESM.tif]

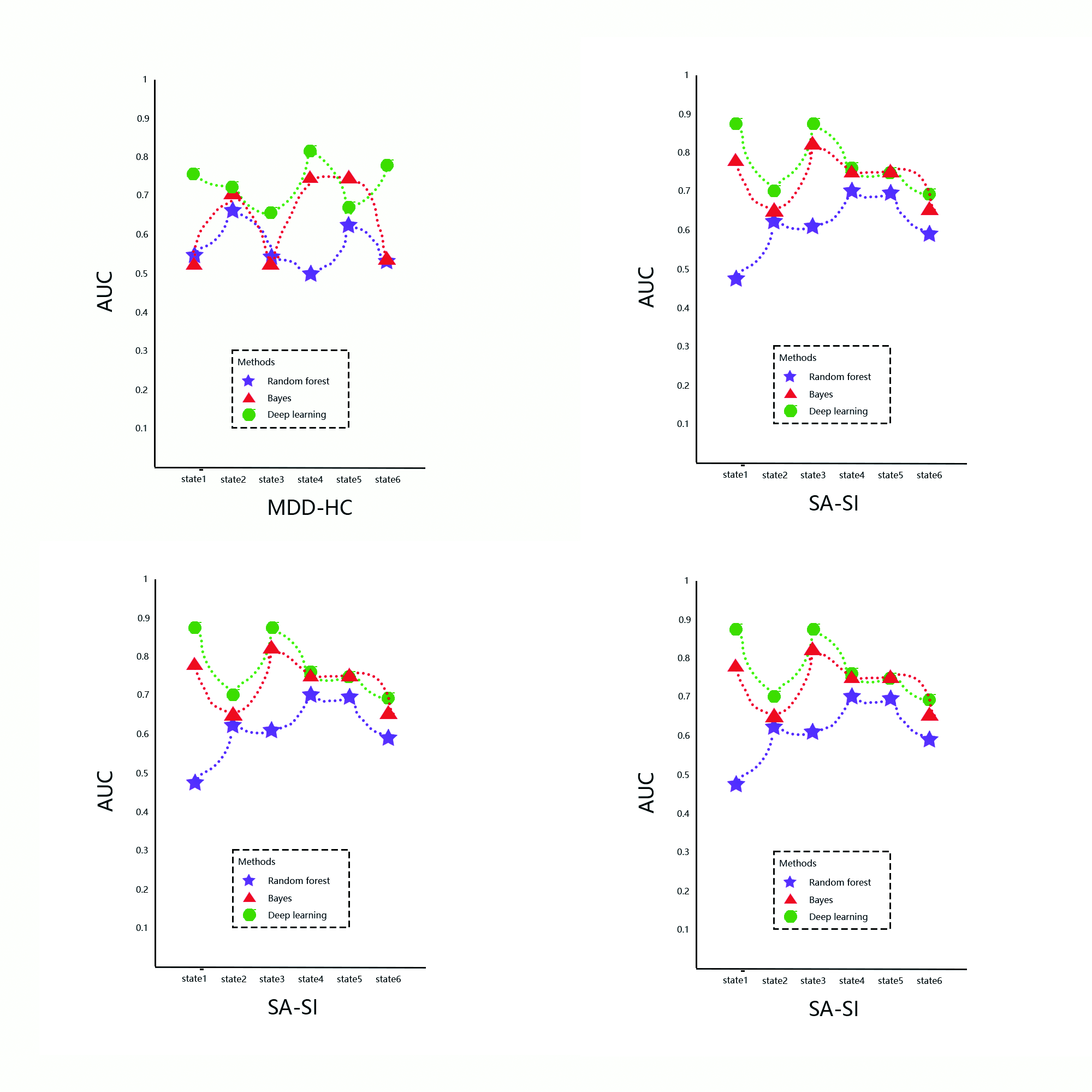

Supplement: Supplementary file 6 — Supplementary Figure 6 [file 41398_2022_2147_MOESM6_ESM.tif]
