# Supplementary material for: Identification of suicidality in patients with major depressive disorder via dynamic functional network connectivity signatures and machine learning
Source: Transl Psychiatry. 2022 Sep 12;12:383. doi: 10.1038/s41398-022-02147-x (PMC9467986; doi:10.1038/s41398-022-02147-x)
Supplement: Supplementary file 7 — Supplementary Table 1 [file 41398_2022_2147_MOESM7_ESM.doc]

| groups | State | Acc | AUC | Sensitivity,  % | Specificity,  % | Cut off Point | Number | acc-*P* |
| --- | --- | --- | --- | --- | --- | --- | --- | --- |
| **SA-HC** | State1 | 66.67 | 0.63 | 0.7 | 0.73 | 0.51 | 40 | 0.119 |
|  | State2 | 61.76 | 0.67 | 0.65 | 0.65 | 0.42 | 2 | 0.19 |
|  | State3 | 83.87 | 0.85 | 0.81 | 0.87 | 0.70 | 28 | ＜0.001* |
|  | State4 | 85.11 | 0.86 | 0.93 | 0.71 | 0.66 | 45 | 0.001* |
|  | State5 | 71.88 | 0.80 | 0.79 | 0.72 | 0.57 | 23 | 0.04* |
|  | State6 | 69.77 | 0.61 | 0.79 | 0.53 | 0.42 | 130 | 0.076 |
| **SI-HC** | State1 | 74.19 | 0.75 | 0.8 | 0.64 | 0.51 | 116 | 0.034* |
|  | State2 | 68.29 | 0.77 | 0.92 | 0.59 | 0.54 | 1 | 0.091 |
|  | State3 | 78.85 | 0.76 | 0.89 | 0.53 | 0.48 | 50 | 0.009* |
|  | State4 | 77.14 | 0.69 | 0.72 | 0.82 | 0.59 | 8 | 0.022* |
|  | State5 | 81.40 | 0.82 | 0.80 | 0.83 | 0.67 | 180 | 0.002* |
|  | State6 | 71.43 | 0.80 | 0.76 | 0.87 | 0.66 | 1 | ＜0.001* |
| **NS-HC** | State1 | 76.92 | 0.82 | 0.8 | 0.73 | 0.58 | 35 | 0.022* |
|  | State2 | 64.71 | 0.62 | 0.65 | 0.65 | 0.42 | 4 | 0.141 |
|  | State3 | 67.65 | 0.73 | 0.63 | 0.8 | 0.51 | 13 | 0.098 |
|  | State4 | 73.17 | 0.81 | 0.79 | 0.76 | 0.61 | 1 | 0.019* |
|  | State5 | 72.22 | 0.73 | 0.78 | 0.67 | 0.52 | 19 | 0.047* |
|  | State6 | 75.56 | 0.73 | 0.87 | 0.53 | 0.46 | 157 | 0.018* |

.
