# Supplementary material for: Identification of suicidality in patients with major depressive disorder via dynamic functional network connectivity signatures and machine learning
Source: Transl Psychiatry. 2022 Sep 12;12:383. doi: 10.1038/s41398-022-02147-x (PMC9467986; doi:10.1038/s41398-022-02147-x)
Supplement: Supplementary file 8 — Supplementary Table 2 [file 41398_2022_2147_MOESM8_ESM.docx]

|  |  | State1 | State2 | State3 | State4 | State5 | State6 |
| --- | --- | --- | --- | --- | --- | --- | --- |
| **Deep**  **learning** | MDD-HC | AUC=0.76  Acc=0.65  Sen=0.59  Spe=0.91 | AUC=0.72  Acc=0.81  Sen=0.89  Spe=0.53 | AUC=0.66  Acc=0.76  Sen=0.79  Spe=0.63 | AUC=0.81  Acc=0.67  Sen=0.63  Spe=0.88 | AUC=0.67  Acc=0.80  Sen=0.93  Spe=0.39 | AUC=0.78  Acc=0.70  Sen=0.67  Spe=0.87 |
|  | SA-SI | AUC=0.87  Acc=0.72  Sen=1  Spe=0.6 | AUC=0.70  Acc=0.70  Sen=0.75  Spe=0.67 | AUC=0.87  Acc=0.81  Sen=0.93  Spe=0.76 | AUC=0.76  Acc=0.79  Sen=0.83  Spe=0.74 | AUC=0.75  Acc=0.76  Sen=0.62  Spe=0.84 | AUC=0.69  Acc=0.71  Sen=0.59  Spe=0.78 |
|  | SA-NS | AUC=0.92  Acc=0.92  Sen=0.89  Spe=0.93 | AUC=0.77  Acc=0.73  Sen=0.75  Spe=0.71 | AUC=0.97  Acc=0.91  Sen=1  Spe=0.84 | AUC=0.84  Acc=0.79  Sen=0.93  Spe=0.63 | AUC=0.80  Acc=0.81  Sen=0.54  Spe=1 | AUC=0.73  Acc=0.74  Sen=0.70  Spe=0.77 |
|  | SI-NS | AUC=0.76  Acc=0.82  Sen=0.84  Spe=0.8 | AUC=0.83  Acc=0.80  Sen=0.74  Spe=0.88 | AUC=0.74  Acc=0.76  Sen=0.78  Spe=0.74 | AUC=0.80  Acc=0.76  Sen=0.73  Spe=0.79 | AUC=0.88  Acc=0.86  Sen=0.79  Spe=0.94 | AUC=0.63  Acc=0.61  Sen=0.4  Spe=0.9 |
| **Bayesian**  **algorithm** | MDD-HC | AUC=0.52  Acc=0.79  Sen=0.96  Spe=0.09 | AUC=0.71  Acc=0.81  Sen=0.90  Spe=0.53 | AUC=0.52  Acc=0.82  Sen=0.97  Spe=0.07 | AUC=0.74  Acc=0.85  Sen=0.90  Spe=0.59 | AUC=0.74  Acc=0.75  Sen=0.75  Spe=0.72 | AUC=0.54  Acc=0.84  Sen=0.95  Spe=0.13 |
|  | SA-SI | AUC=0.78  Acc=0.83  Sen=0.6  Spe=0.95 | AUC=0.65  Acc=0.68  Sen=0.47  Spe=0.83 | AUC=0.82  Acc=0.85  Sen=0.75  Spe=0.89 | AUC=0.75  Acc=0.75  Sen=0.8  Spe=0.70 | AUC=0.75  Acc=0.79  Sen=0.57  Spe=0.92 | AUC=0.66  Acc=0.70  Sen=0.5  Spe=0.83 |
|  | SA-NS | AUC=0.82  Acc=0.84  Sen=0.7  Spe=0.93 | AUC=0.65  Acc=0.65  Sen=0.59  Spe=0.71 | AUC=0.83  Acc=0.83  Sen=0.81  Spe=0.84 | AUC=0.81  Acc=0.81  Sen=0.83  Spe=0.79 | AUC=0.87  Acc=0.88  Sen=0.79  Spe=0.94 | AUC=0.69  Acc=0.69  Sen=0.61  Spe=0.77 |
|  | SI-NS | AUC=0.8  Acc=0.8  Sen=0.8  Spe=0.8 | AUC=0.70  Acc=0.71  Sen=0.75  Spe=0.65 | AUC=0.72  Acc=0.77  Sen=0.86  Spe=0.58 | AUC=0.75  Acc=0.74  Sen=0.74  Spe=0.75 | AUC=0.76  Acc=0.77  Sen=0.8  Spe=0.72 | AUC=0.69  Acc=0.70  Sen=0.76  Spe=0.63 |
| **Random**  **forest** | MDD-HC | AUC=0.57  Acc=0.80  Sen=0.96  Spe=0.18 | AUC=0.68  Acc=0.83  Sen=0.95  Spe=0.41 | AUC=0.54  Acc=0.80  Sen=0.94  Spe=0.13 | AUC=0.5  Acc=0.83  Sen=0  Spe=1 | AUC=0.62  Acc=0.8  Sen=0.96  Spe=0.28 | AUC=0.53  Acc=0.88  Sen=1  Spe=0.07 |
|  | SA-SI | AUC=0.48  Acc=0.6  Sen=0.1  Spe=0.85 | AUC=0.62  Acc=0.63  Sen=0.53  Spe=0.71 | AUC=0.61  Acc=0.75  Sen=0.25  Spe=0.97 | AUC=0.70  Acc=0.70  Sen=0.8  Spe=0.59 | AUC=0.69  Acc=0.74  Sen=0.5  Spe=0.88 | AUC=0.59  Acc=0.62  Sen=0.43  Spe=0.76 |
|  | SA-NS | AUC=0.72  Acc=0.76  Sen=0.5  Spe=0.93 | AUC=0.68  Acc=0.68  Sen=0.53  Spe=0.82 | AUC=0.81  Acc=0.83  Sen=0.69  Spe=0.95 | AUC=0.71  Acc=0.72  Sen=0.8  Spe=0.63 | AUC=0.77  Acc=0.78  Sen=0.71  Spe=0.83 | AUC=0.69  Acc=0.69  Sen=0.71  Spe=0.67 |
|  | SI-NS | AUC=0.79  Acc=0.8  Sen=0.85  Spe=0.73 | AUC=0.79  Acc=0.73  Sen=0.83  Spe=0.59 | AUC=0.75  Acc=0.79  Sen=0.86  Spe=0.63 | AUC=0.63  Acc=0.63  Sen=0.67  Spe=0.58 | AUC=0.67  Acc=0.70  Sen=0.84  Spe=0.5 | AUC=0.57  Acc=0.61  Sen=0.80  Spe=0.33 |
